# Associations between cognitive screening performance and motor symptoms in Parkinson’s disease: a systematic review and meta-analysis

**DOI:** 10.1590/1980-5764-DN-2023-0102

**Published:** 2024-09-02

**Authors:** Karlee Patrick, Elizabeth Cousins, Mary Beth Spitznagel

**Affiliations:** 1 Kent State University, College of Arts and Sciences, Department of Psychological Sciences, Kent, Ohio, USA.

**Keywords:** Parkinson’s Disease, Mental Status and Dementia Tests, Movement Disorders, Doença de Parkinson, Testes de Estado Mental e Demência, Transtornos dos Movimentos

## Abstract

Although the most prominent symptoms of Parkinson’s disease (PD) are those impacting movement, cognitive dysfunction is prevalent and often presents early in the disease process. Individuals with cognitive symptoms of PD often complete cognitive screening, making it important to identify factors associated with cognitive screening performance to ensure prompt and accurate detection of cognitive impairments.

**Objective:**

Despite a body of research examining relationships between motor symptoms and cognitive dysfunction in PD, no prior study has undertaken a systematic review of the magnitude of the relationship between motor symptoms and cognitive screening performance in PD.

**Methods:**

This study was a systematic review and meta-analysis of the relationship between cognitive screening performance, as assessed by the Montreal Cognitive Assessment (MoCA), and motor symptoms of PD. After the systematic screening, 20 studies were included, and meta-regressions using mixed-effects models were conducted.

**Results:**

Motor symptoms across included studies were relatively mild, but average MoCA scores were at the established cutoff for risk of dementia in PD. The average disease duration was 5 years. Consistent with hypotheses, more severe motor symptoms were associated with lower MoCA scores (*r=*-0.22 (95%CI -0.29 to -0.16), p<0.001), indicating worse cognitive functioning.

**Conclusion:**

The results indicate a significant negative correlation between MoCA performance and motor symptoms of PD. Average MoCA scores captured early disease-stage cognitive impairment when motor symptoms remained relatively mild. Serial screening for cognitive impairment beginning early in the disease course may be of benefit to ensure that cognitive dysfunction is detected as it arises.

## RESUMO

Embora os sintomas mais proeminentes da doença de Parkinson (DP) sejam aqueles que afetam o movimento, a disfunção cognitiva é prevalente e muitas vezes se apresenta no início do processo da doença. Indivíduos com sintomas cognitivos de DP frequentemente realizam triagem cognitiva, tornando importante identificar os fatores associados ao desempenho da triagem cognitiva para garantir a detecção rápida e precisa de deficiências cognitivas.

**Objetivo:**

Apesar de um conjunto de pesquisas examinar as relações entre sintomas motores e disfunção cognitiva na DP, nenhum trabalho anterior realizou uma revisão sistemática da magnitude da relação entre sintomas motores e desempenho na triagem cognitiva na DP.

**Métodos:**

O presente estudo foi uma revisão sistemática e meta-análise da relação entre o desempenho da triagem cognitiva, avaliada pela Avaliação Cognitiva de Montreal (*Montreal Cognitive Assessment* – MoCA), e os sintomas motores da DP. Após triagem sistemática, 20 estudos foram incluídos e foram realizadas meta-regressões utilizando modelos de efeitos mistos.

**Resultados:**

Os sintomas motores nos estudos incluídos foram relativamente leves, mas as pontuações médias do MoCA estavam no ponto de corte estabelecido para o risco de demência na DP. A duração média da doença foi de 5 anos. Consistente com as hipóteses, sintomas motores mais graves foram associados a pontuações mais baixas no MoCA, r=-0,22 (IC95% -0,29 to -0,16), p<0,001), indicando pior funcionamento cognitivo.

**Conclusão:**

Os resultados indicam uma correlação negativa significativa entre o desempenho no MoCA e os sintomas motores da DP. As pontuações médias do MoCA capturaram o comprometimento cognitivo em estágio inicial da doença, quando os sintomas motores permaneceram relativamente leves. O rastreio em série do comprometimento cognitivo que começa no início do curso da doença pode ser benéfico para garantir que a disfunção cognitiva seja detectada à medida que surge.

## INTRODUCTION

### Overview of Parkinson’s disease

Parkinson’s disease (PD) is a progressive neurological disorder characterized by resting tremor, bradykinesia, rigidity, impaired postural reflex, and instability^1^. Motor symptoms are the most commonly identified symptoms of PD^2,3^. PD involves progressive loss of dopaminergic neurons in the substantia nigra and projections to the striatum^3^, leading to generalized slowing, reduced initiation of intentional movements, stooped posture, reduced arm swing, shuffling steps, facial masking, resting tremor, cogwheel rigidity, and bradykinesia^2^. However, these motor symptoms may not be observable until individuals have lost 50–80% of dopaminergic neurons^2^. As such, other symptoms of PD (e.g., cognitive dysfunction) often occur before the onset of motor symptoms.

Although the most prominent symptoms of PD are those impacting movement^1,2^, cognitive dysfunction is a prevalent symptom of this disease that has determinantal consequences for the quality of life and prognosis for these individuals^3-5^. Approximately 20–25% of individuals with PD are diagnosed with mild cognitive impairment^6^. Lifetime prevalence of dementia in the context of PD increases with age, and up to 80% of individuals with PD are diagnosed with dementia within 20 years of PD diagnosis^6^. Cognitive testing in PD reveals a range of severity across domains of cognitive impairment, including executive functions, visuospatial abilities, psychomotor speed, memory, language, and verbal fluency^7-9^. Decreased dopamine levels in the brain in PD may be a driving factor in cognitive impairment, as dopamine dysfunction is associated with impairments across several cognitive domains^10-12^. Due to high rates of cognitive dysfunction in this population, individuals with PD often complete cognitive screening assessments^9,13,14^. Previous research suggests that motor symptoms may be associated with performance on cognitive testing^10-12^. However, a few prior research has explored whether performance on brief, cognitive screening measures is associated with motor symptoms of PD. This is important, given that cognitive screening measures are often used to determine whether an individual with PD is struggling with cognitive impairment and referred for formal neuropsychological assessment^5^. In an effort to enhance the utility of cognitive screening in PD, it is essential to understand the extent to which motor symptoms of PD, the most prominent and commonly identified PD symptom, are associated with cognitive screening performance.

In addition to motor symptoms of PD, several individual factors including disease duration, age, race, and biological sex could be involved in associations between motor symptoms and cognitive screening performance. For example, males are more likely to develop PD than females at a rate of 1.5 to 1^15^, and Caucasian individuals show higher prevalence compared with other racial or ethnic groups^16^. Older age itself is associated with greater cognitive dysfunction^17,18^, and motor symptoms of PD typically worsen with a longer duration of disease and older age^19^. Examination of these individual differences is thus essential when considering associations between motor symptoms and cognitive screening performance.

Assessing the extent to which motor symptoms are associated with cognitive screening performance in individuals with PD, and if this association differs based on individual differences, may help improve accurate and prompt detection of cognitive dysfunction in this population^11,12,20,21^. Despite a body of research examining relationships among motor symptoms and cognitive dysfunction in PD^22^, no prior study has undertaken systematic review and meta-analysis to determine the magnitude of relationships among motor symptoms and performance on cognitive screening measures. It is hypothesized that more severe motor symptoms will be associated with lower cognitive screening performance. In light of the heterogeneity of disease course and treatment, greater prevalence of PD in males than females, and possible sex differences in presentations of cognitive dysfunction^15,16,21^, this study will also explore the influence of individual factors in moderating these relationships.

## METHODS

The following procedures were pre-registered on PROSPERO in March 2023 (ID: CRD42023415130). This review follows PRISMA^23^ guidelines.

### Search strategy

A search of articles published since 2005 was conducted on PubMed, CINHAL, Medline, and PsychInfo in June 2023. The Montreal Cognitive Assessment (MoCA) is considered the gold standard for cognitive screening in individuals with PD^5,21^. The MoCA was validated in 2005^24^; thus, 2005 was chosen as the earliest publication year included in this review. The following search terms were used to broadly capture relevant articles: PD and MoCA. A filter was also applied to select articles available in English, peer-reviewed articles, and participants older than 18 years of age. Age was not otherwise restricted due to a range of age of onset in PD.

### Study selection

Article information from database searches was compiled into Covidence, which is a screening and data extraction tool for conducting systematic reviews. Two reviewers completed title/abstract and full-text reviews to determine the eligibility according to the criteria. Disagreements were resolved via discussion. The inclusion criteria were as follows:

- The study sample consisted of individuals with formally diagnosed PD not secondary to other conditions or medications based on established criteria or physician diagnosis.
- The MoCA was employed to measure cognitive screening performance.
- The study measured motor symptoms of PD as continuous (or quasi-continuous) variables.

The exclusion criteria were as follows:

- The study sample included individuals with parkinsonism secondary to other conditions or medications.
- The study sample included individuals with Alzheimer’s disease, Lewy body dementia, mixed dementia, or other cognitive impairments not associated with PD (i.e., only individuals with PD-associated cognitive impairment were included in the current meta-analysis).
- The article specified the risk of PD, but participants had not been formally diagnosed.
- The MoCA was not administered.
- Only post-treatment (e.g., medication trial and deep brain stimulation) data were available.
- The article was a follow-up study with no baseline data, or baseline data were based on a retrospective report.
- The article was a non-human animal study.
- The article was not peer-reviewed.
- Study outcomes did not include sufficient data to be extracted.

Exclusion criteria were documented in Covidence as well. Regarding study design, cross-sectional studies were included. In the case of a longitudinal design, the correlation coefficient from the first time point was used. Case-control designs were not excluded, and the correlation within the cases relevant to the study (individuals with PD) was used. Randomized clinical trials/intervention studies were included, and baseline (i.e., prior to treatment) data were used if provided. Review articles, articles with non-original data, and articles with study populations drawn from the same database of participants within the same inclusion years as other articles in this review were excluded.

### Data extraction

Two independent reviewers double-entered data from included studies, and data entry forms were compared for accuracy. Disagreements were resolved via discussion. The following information was extracted: instrument information and statistics for relevant measures (i.e., MoCA and motor symptoms of PD), study design, study location, participant information (i.e., age, sex, race, and education), description of the sample (e.g., older/younger onset of PD and age of disease onset), formal diagnosis (PD) and method of diagnosis, and current medical treatment for PD in the sample. When possible, correlations between the MoCA and motor symptoms were extracted from the text of the included articles. If correlations among variables were not included in the text of articles, or the study did not otherwise provide sufficient data for effect size estimation, the corresponding authors of these articles were contacted via email with requests to provide the necessary data for inclusion. If the corresponding author did not provide the required information before the completion of this review, the study was excluded.

The Appraisal tool for Cross-Sectional Studies (AXIS), which is a tool consisting of 20 components, was used to rate and evaluate bias risk for each included study^25^. Each study was evaluated by the same independent reviewers.

### Data synthesis

Data analysis was conducted using R and RStudio. Using the R packages “metasens” and “meta,” meta-regressions using mixed effects models^26,27^ were conducted to estimate meta-correlations between MoCA performance and motor symptoms. A minimum of 10 studies was required for each meta-correlation^26^. For meta-regressions with categorical moderators, a minimum of 10 studies per category was also required^28^. Heterogeneity between effect sizes was investigated using the *Q* or *I* statistic and a forest plot^29^. A funnel plot was conducted to evaluate for publication bias.

## RESULTS

**Figure 1:**
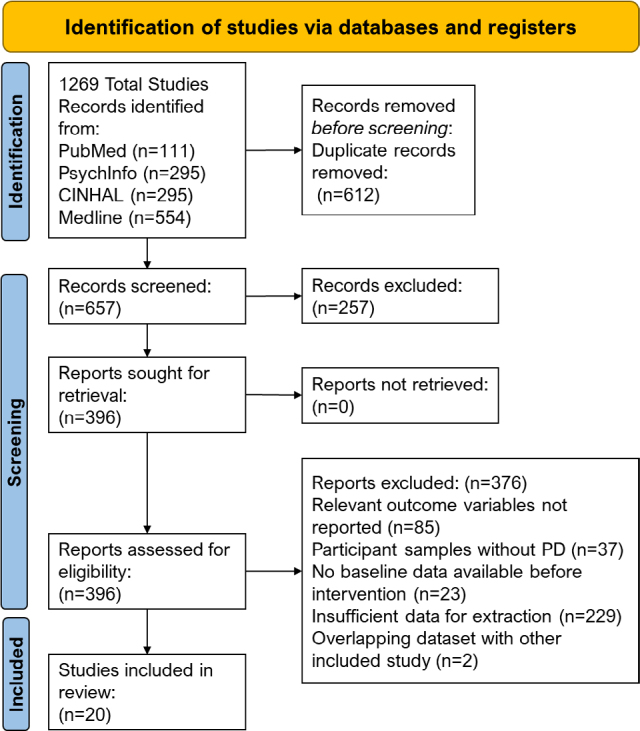
PRISMA flow diagram.

A systematic review search using PubMed, Medline, PsychInfo, and CINHAL yielded 1,269 studies. After removing duplicates, 657 studies remained to be screened under title-abstract review. At this stage, 257 studies that did not include individuals with PD and/or assess cognition using the MoCA were removed. A total of 396 studies were assessed under full-text review, 376 studies were excluded at full-text review, and 20 studies were included in the final analyses (Figure 1). Studies were excluded at full-text review due to the following: Did not include relevant outcome variables (i.e., motor symptoms, n=85), included participants with PD secondary to other conditions or medications (n=37), study design precluded inclusion (e.g., longitudinal design without baseline data; n=23), dataset overlapped with another included study (n=2), or insufficient data for extraction due to lack of results included in text or provided by the corresponding author upon request (n=229). Corresponding authors of the 20 studies included in the final meta-analysis responded via email with the necessary correlations and participant data.

### Study descriptives

#### Study designs and locations

Descriptive information and a summary of included studies can be found in Table 1 ^30-49^. Most study designs were cross-sectional (n=13), with a small number of case-control (n=4), intervention (n=2), and longitudinal (n=1) designs. Included studies were conducted in a variety of locations, including Europe (n=5), Asia (n=4), the United States of America (n=4), Latin or South America (n=3), Oceania (n=2), and a combination of multiple international sites (n=2).

**Table 1:** Summary of included studies.

Study	Country	Study design	N	Age Mean (SD)	MoCA Mean (SD)	UPDRS III Mean (SD)	r(MoCA and UPDRS III)
Aiello et al.,^30^	Europe	Cross sectional	73	67.0 (8.9)	22.2 (4.7)	13.2 (9.2)	-0.32
Baik et al.,^31^	Asia	Intervention (Baseline)	50	67.9 (7.1)	22.4 (4.2)	20.0 (7.1)	-0.30
Chaudhary et al.,^32^	Asia	Cross sectional	64	60.0 (6.1)	23.8 (3.3)	13.0 (6.5)	-0.31
Flannery et al.,^33^	Oceania	Case control	50	68.5 (7.6)	25.8 (3.8)	30.3 (13.2)	-0.50
Hendershot et al.,^34^	USA	Cross sectional	82	67.2 (8.4)	24.7 (4.8)	35.3 (11.8)	-0.40
Hoops et al.,^35^	USA	Cross sectional	132	65.1 (9.7)	25.0 (3.8)	24.6 (11.3)	-0.24
Chen et al.,^36^	Multiple sites	Longitudinal correlation (Baseline)	232	60.3 (9.2)	28.2 (1.3)	18.4 (8.4)	-0.11
Kahya et al.,^37^	USA	Case control	24	68.0 (N/A)	27.58 (1.52)	31.29 (11.25)	-0.04
Neikrug et al.,^38^	USA	Cross sectional	80	67.4 (8.8)	24.6 (3.4)	3.5 (2.3)	0.08
Pimenta et al.,^39^	Latin + South America	Cross sectional	58	69.0 (N/A)	17.2 (4.7)	32.3 (12.3)	-0.13
Prell et al.,^40^	Europe	Cross sectional	52	74.4 (6.6)	25.0 (3.0)	29.3 (12.2)	-0.10
Reginold et al.,^41^	Multiple sites	Cross sectional	490	71.3 (5.2)	25.6 (3.1)	27.5 (13.9)	-0.20
Rong et al.,^42^	Asia	Cross sectional	66	65.1 (6.0)	21.6 (0.54)	32.3 (1.8)	-0.35
Rucco et al.,^43^	Europe	Case control	31	65.0 (8.2)	22.4 (3.3)	25.0 (9.6)	-0.01
Silverdale et al.,^44^	Europe	Cross sectional	1556	68.0 (9.5)	25.1 (3.6)	26.7 (13.6)	-0.24
Soares et al.,^45^	Latin + South America	Cross sectional	81	63.0 (9.8)	23.7 (4.8)	44.8 (15.0)	-0.22
Stern et al.,^46^	Latin + South America	Cross sectional	74	67.5 (8.7)	24.6 (3.0)	10.0 (8.6)	-0.10
Still et al.,^47^	Oceania	Cross sectional	19	68.8 (6.5)	26.1 (2.7)	22.2 (10.8)	-0.47
Tandra et al.,^48^	Asia	Non-randomized intervention (Baseline)	40	55.5 (9.8)	28.6 (1.8)	39.7 (10.1)	-0.44
Thomas et al.,^49^	Europe	Case control	100	64.5 (7.7)	28.0 (2.0)	22.6 (11.7)	-0.11

Abbreviations: SD, standard deviation; N/A, not available.

#### Assessment of cognitive dysfunction

All included studies used the 30-point full version of the MoCA^24^, and the total MoCA scores out of 30 points were used in analyses. The MoCA assesses a range of cognitive domains, is extensively validated in individuals with various levels of cognitive impairment, shows high sensitivity and specificity in the initial detection of cognitive dysfunction in PD, and is the most widely used screening measure in PD^5,6,21^.

#### Assessment of motor symptoms

Motor symptoms were assessed using the MDS-Unified Parkinson’s Disease Rating Scale Part III (UPDRS III)^50^ in all included articles. The UPDRS III combines assessment of the following motor symptoms into one final score: speech, facial expression, rigidity of the neck, arms, and legs, finger tapping, hand movements, toe-tapping, leg agility, arising from a chair, gait, posture, postural stability, body bradykinesia, postural hand tremor, kinetic hand tremor, and resting tremor amplitude and constancy^50^. Total scores range from 0 to 132, with higher scores indicating worse motor symptoms^50^. Scores range from 0 to 4 on each item, and 33 scores are summed to obtain a total score^50^.

#### Participant demographics and descriptive statistics

The average age of participants was 66.5 (SD=4.1) years. The average disease duration was 5.1 (SD=2.5) years. Participants had 14.4 (SD=2.4) years of education on average. Study samples largely included more male than female participants, averaging 61.9% (SD=10.1) males across samples. The mean MoCA score was 24.8 (SD=5.2). The mean UPDRS III score was 23.7 (SD=12.1). Participants’ race or ethnicity cannot be summarized, as only one included study reported participant race.

### Analyses of heterogeneity

**Figure 2:**
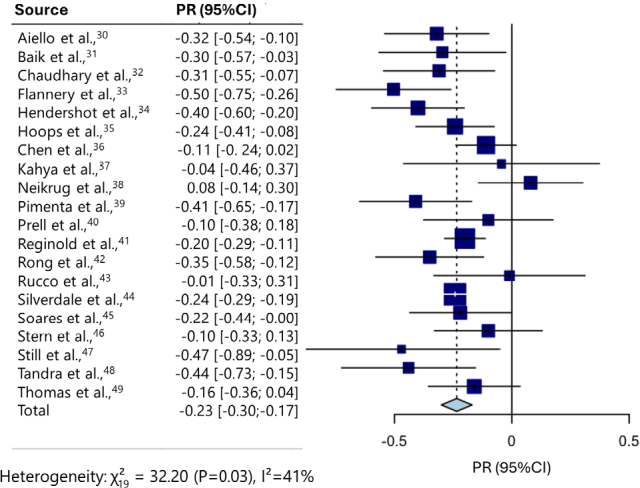
Forest plot of included studies.

**Figure 3:**
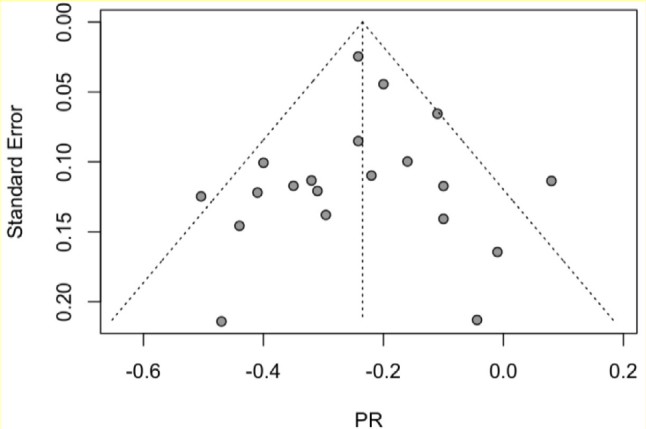
Funnel plot of included studies.

A fixed-effects model demonstrated moderate heterogeneity (I^2^=37.8%, *Q*(19)=30.89, p<0.05, H=1.27). Given significant heterogeneity results, a random-effects model^28^ was conducted to estimate the meta-correlation between the MoCA and motor symptoms. A forest plot (Figure 2 ^30-49^) and funnel plot (Figure 3) were used to confirm significant heterogeneity.

### Meta-correlations

#### Motor symptoms and MoCA performance

A random-effects model found that the meta-correlation of motor symptoms and MoCA performance was *r=*-0.22 (95%CI -0.29 to -0.16), p<0.001). The estimate of the standard deviation of the distribution of true effect sizes was τ = 0.07.

### Moderation analyses

#### Motor symptoms and MoCA performance

Meta-regression models using random intercepts showed no significant moderation of the relationship between motor symptoms and MoCA performance ratings based on age, education, disease duration, or percent of the sample identifying as male. Most included studies, with only two exceptions, assessed participants’ motor symptoms and cognition while participants were taking their medication for PD (all studies specified dopamine agonist, levodopa, or L-dopa equivalent daily dose) as prescribed (ON state of medication use). Thus, medication use in the ON versus OFF state in individuals with PD could not be included as a moderator in the current analyses. Race could not be explored as a moderator, as only two of the included studies reported participant race and/or ethnicity. No included studies assessed individuals with early-onset PD, so the age of onset group could not be assessed as a moderator. The number of studies reporting the average age of symptom onset was also insufficient for this variable to be included in analyses.

### Publication bias and study quality assessment

Examination of the funnel plot of included studies (Figure 3) indicated a fairly symmetrical distribution of effect sizes. This observation was supported by a nonsignificant Egger’s test of asymmetry (t=0.1, df (18), p=0.95). AXIS ratings for included studies can be found in Appendix A. Many studies were missing basic demographic information and descriptive statistics, limiting the generalizability and quality of study results. Additionally, a priori or post hoc power analyses were not conducted for most included studies.

## DISCUSSION

This study sought to determine the meta-correlation between motor symptoms of PD and cognitive function. Supporting hypotheses, worse ratings of motor symptoms (higher scores on the UPDRS III) were associated with poorer cognitive screening performance (lower MoCA scores). The meta-correlation was significant and negative (*r=*-0.22), though small in effect. Mean UPDRS III scores indicated mild motor symptoms (M=23.7) based on prior research investigating UPDRS III motor symptom severity cutoff values^51^. It is possible that the correlation between motor symptoms and MoCA performance would be stronger in individuals with more severe motor symptoms, as range restriction in this study may have limited the strength of this relationship. Future research should explore whether the association between motor symptoms and cognitive dysfunction differs when motor symptoms are more severe, as prior research indicates that more severe motor symptoms are associated with the risk of dementia in PD^10^. However, identifying that even mild motor symptoms are significantly associated with cognitive dysfunction is important, given that cognitive screening may be conducted earlier in the disease course when motor symptoms are less severe than in later stages^22^.

The meta-analysis revealed that motor symptoms in the included studies were relatively mild, but mean MoCA scores (M=24.8) were at the established cutoff for risk of dementia in PD (cutoff = total score <25 out of 30)^52^. The average disease duration in this study was 5 years. Together, this suggests that MoCA scores captured early disease-stage cognitive impairment when motor symptoms remained relatively mild. This finding supports prior research indicating that cognitive deficits may be present early in the disease course, occurring prior to the worsening of motor symptoms^10^. Overall, the results indicate that screening for cognitive impairment using the MoCA should begin early in the disease course and be repeated over time to ensure that cognitive dysfunction is detected as it arises.

The MoCA is the most commonly used measure for screening of cognitive dysfunction in PD^5,21^, making it important to identify the consistency of associations between motor symptoms and MoCA performance in this population. MoCA scores are often used to determine whether an individual with PD is referred for formal neuropsychological assessment, which can help identify specific cognitive dysfunction and inform treatment recommendations^5^. Identifying the extent to which motor symptoms are associated with MoCA performance can help ensure accurate and prompt cognitive screening in PD and subsequent treatment planning, even during disease stages when motor symptoms remain mild. To date, very few studies have reported associations between cognitive screening performance and motor symptoms. One recent study found that motor symptoms were related to memory, executive function, language, and visuospatial functions^22^; however, this study neither assessed cognitive screening performance nor included details on how cognitive domains were assessed. Others have found significant associations between the Mini-Mental State Examination (MMSE) and motor symptoms^11,53^. However, because the MoCA is more commonly used, shows superior validity, and is better suited to assessing decline across cognitive domains in PD compared with the MMSE^54^, it is likely that the MoCA will continue to be considered the gold standard.

It is possible that cognitive impairments observed in PD result from motor symptoms influencing test performance directly (i.e., via motor slowing or tremor). However, individuals with PD show cognitive impairments even when time and motor constraints are removed, and motor symptoms were not associated with performance on a brief cognitive screening measure in prior research^6,13^. Given these past findings and the relatively mild motor symptoms in the current meta-analysis, it is unlikely that the negative relationship observed between motor symptoms and MoCA performance results from motor symptoms influencing test performance directly.

The association between motor symptoms and MoCA performance was not moderated by age, education, disease duration, or percent of the sample identifying as male. It is possible that range restriction limited the full exploration of the influence of age, as the average age of participants was 67 years with a standard deviation of only 4 years. No studies in the current meta-analysis examined individuals with early-onset PD. Early-onset PD is typically associated with more severe involuntary muscle movements at disease onset compared with older-onset PD^55^. The findings from this study may thus not generalize to individuals with an early-onset diagnosis. In addition, though dementia is less common, approximately one-third of individuals with early-onset PD meet the criteria for mild cognitive impairment^55^. Assessment of whether the associations between motor symptoms and cognitive dysfunction differ in individuals with early-onset versus older-onset PD would be of benefit in future research. Participant sex did not also influence the relationship between motor symptoms and cognitive dysfunction. These findings highlight the importance of assessing motor symptoms and cognitive dysfunction early in the disease course for both males and females with PD.

Despite the strengths of this study, several limitations must be noted. Most studies included did not report correlations among variables in the study text, and many relevant articles could not be included due to insufficient data for extraction. In addition, though studies were diverse in terms of geographic location, neither race nor ethnicity of study samples could be explored as a moderator, as only two of the included studies reported participant race and/or ethnicity. To the extent possible, future research should explore whether associations between motor symptoms and MoCA performance are present to the same degree in diverse populations. In addition, participants across studies were highly educated, with over 14 years of education on average. Prior research shows associations between higher educational attainment and performance on global cognitive screening measures in individuals with PD^56^. Future research should explore links between motor symptoms and cognitive dysfunction in populations with lower education levels to ensure the generalizability of findings. Though beyond the scope of the current study, the included articles largely did not assess genetic polymorphisms that are commonly associated with PD. Prior research suggests that different polymorphisms are associated with heterogeneous patterns of motor symptoms^57^ and cognitive dysfunction^58^. Future research could also explore whether relationships between motor symptoms and cognitive dysfunction differ based on genetic factors. This study also could not explore the influence of common PD medications on motor symptoms and their association with cognitive dysfunction. This is important, given that motor symptoms of PD often fluctuate with medication use. Eighteen of the 20 studies included in this meta-analysis assessed individuals while they were taking their medications as prescribed (ON state of medication use). Examination of the influence of medication use on associations between motor symptoms and MoCA performance could not be explored due to the small sample size (n=2) of studies including individuals who were not currently taking their medications.

The current systematic review and meta-analysis sought to investigate associations between motor symptoms and cognitive screening performance with the goal of improving detection of cognitive dysfunction, and subsequently, treatment and quality of life for individuals with PD. The average MoCA scores were at cutoffs for risk of dementia across studies, suggesting that cognitive screenings should begin early in PD in the context of relatively mild motor symptoms. The findings represent a step toward understanding the magnitude and consistency of the relationship between motor symptoms and MoCA performance. Future research should explore findings in individuals with more severe motor symptoms and in more diverse samples to ensure the generalizability of findings.
